# Supplementary material for: Transcriptome assembly at single-cell resolution with Beaver
Source: Bioinformatics. 2025 Jul 15;41(Suppl 1):i323–31. doi: 10.1093/bioinformatics/btaf236 (PMC12261439; doi:10.1093/bioinformatics/btaf236)
Supplement: btaf236_Supplementary_Data [file btaf236_supplementary_data.pdf]

Supplementary Materials for
“Transcriptome Assembly at Single-Cell Resolution with Beaver”

Qian Shi[1], Qimin Zhang[1], and Mingfu Shao[1,2,*]

[1]Department of Computer Science and Engineering, School of Electronic Engineering and Computer Science, The Pennsylvania State University
[2]Huck Institutes of the Life Sciences, The Pennsylvania State University

January 23, 2025

List of Supplementary Notes

1 PseudoCode for Candidate Transcript Generation . . . 3
2 Features Description . . . 4

List of Supplementary Figures

1 Top 20 Feature Correlations with General and Cell-Specific Labels in the HEK293T Dataset . . . 6
2 Top 20 Feature Importance for Beaver-General in the HEK293T Dataset . . . 7
3 Top 20 Feature Correlations with General and Cell-Specific Labels in the HEK293T-Sim Dataset 8
4 Top 20 Feature Importance for Beaver-General in the HEK293T-Sim Dataset. . . . 9
5 Top 20 Feature Importance for Beaver-Specific in the HEK293T-Sim Dataset. . . . 10
6 Comparison of “General” versus “Cell-Specific” matching counts across assemblers. . . . 11
7 Beaver vs. TransMeta on HEK293T dataset. . . . 12
8 Beaver vs. PsiCLASS on HEK293T dataset. . . . 12
9 Beaver vs. Aletsch on HEK293T dataset. . . . 13
10 Beaver vs. StringTie2 on HEK293T dataset. . . . 13
11 Beaver vs. Scallop2 on HEK293T dataset. . . . 14
12 Beaver vs. TransMeta on Mouse-Fibroblast dataset. . . . 14
13 Beaver vs. PsiCLASS on Mouse-Fibroblast dataset. . . . 15
14 Beaver vs. Aletsch on Mouse-Fibroblast dataset. . . . 15
15 Beaver vs. StringTie2 on Mouse-Fibroblast dataset. . . . 16
16 Beaver vs. Scallop2 on Mouse-Fibroblast dataset. . . . 16
17 Beaver vs. TransMeta on HEK293T-Sim dataset. . . . 17
18 Beaver vs. PsiCLASS on HEK293T-Sim dataset. . . . 17
19 Beaver vs. Aletsch on HEK293T-Sim dataset. . . . 18
20 Beaver vs. StringTie2 on HEK293T-Sim dataset. . . . 18
21 Beaver vs. Scallop2 on HEK293T-Sim dataset. . . . 19
22 Beaver vs. TransMeta on Mouse-Fibroblast-Sim dataset. . . . 19
23 Beaver vs. PsiCLASS on Mouse-Fibroblast-Sim dataset. . . . 20
24 Beaver vs. Aletsch on Mouse-Fibroblast-Sim dataset. . . . 20
25 Beaver vs. StringTie2 on Mouse-Fibroblast-Sim dataset. . . . 21
26 Beaver vs. Scallop2 on Mouse-Fibroblast-Sim dataset. . . . 21

*Correspondence should be addressed to mxs2589@psu.edu.

List of Supplementary Tables

1 Description of HEK293T and Mouse-Fibroblast datasets. 22

Supplementary Note 1: PseudoCode for Transcript Generation

Algorithm 1 Dynamic Programming for Candidate Transcript Generation

Input: Connected component $G_c = (V_c, E_c)$ ▷ Connected components n transcript fragment graph
Output: Set of optimal paths P_c ▷ Each path represents a candidate transcript

function FindOptimalPaths(G_c)
  $(v_1, v_2, \ldots, v_{|V_c|}) \leftarrow$ TopologicalSort(G_c) ▷ $O(|V_c| + |E_c|)$
  paths[v] $\leftarrow$ new MinHeap() for all $v \in V_c$ ▷ Initialize Min-Heaps
  for $j \leftarrow 1$ to $|V_c|$ **do**
    paths[v_i].push{(MergingScore(v_i), v_i} ▷ Initialize single-vertex path
    for $(v_i, v_j) \in E_c$ **do** ▷ Check incoming edges to v_j
      for each path p in paths[v_i].getAll() **do** ▷ $O(p_n)$
        new_path $\leftarrow p \cup \{v_j\}$ ▷ Extend path with new vertex
        score $\leftarrow$ MergingScore(new_path)
        if paths[v_j].size() $< p_n$ **then** ▷ Limit p_n paths stored per node
          paths[v_j].push(score, new_path)
        else if score $>$ paths[v_j].top().score **then** ▷ Heap is full
          paths[v_j].pop() ▷ Remove lowest scoring path in the Min-Heap
          paths[v_j].push(score, new_path)
        end if
      end for
    end for
  end for
  return $P_c \leftarrow$ TopK($\bigcup_{v \in V_c} \{p \mid (\text{score}, p) \in \text{paths}[v].\text{getAll}()\}, p_c$) ▷ Limit p_c paths stored per graph
end function
function MergingScore(path p)
  $b \leftarrow \min_{j \in J(p)}$ JunctionWeight(j, p) ▷ Bottleneck weight
  $n \leftarrow |J(p)|$ ▷ Number of junctions
  return $b \cdot n$
end function
function JunctionWeight(junction j, path p)
  $X_j \leftarrow \{t \mid t$ contains j and is compatible with $p\}$ ▷ Find compatible transcripts
  return $\sum_{t \in X_j}$ coverage(t) ▷ Sum coverage of supporting transcripts
end function

Supplementary Note 2: Features Description

This note describes the feature sets used to train Beaver-General and Beaver-Specific. We developed two feature sets: (1) general features (n=30) that assess overall transcript reliability across the dataset, and (2) cell-specific features (n=21) that evaluate expression likelihood in individual cells. These features are computed for every unique pair of candidate path p and cell c in the transcript fragment graphs, with general features being replicated across all cells for the same p.

(Shown in *Italic* are the headers of the Beaver's feature table.)

General Features ($n = 30$)

- *sample_size*: Total number of input cells/samples.
- *bottleneck_coverage*: Bottleneck junction-score $BJ(p)$ from the dynamic programming solution.
- *highest_coverage*: Maximum coverage value observed among raw transcript fragments $t \sim p$.
- *num_junctions*: Total number of junctions in path p, i.e. $NJ(p)$.
- Compatible junction coverage:

  (*min_comp_cov*, *max_comp_cov*, *median_comp_cov*, *mean_comp_cov*, *std_comp_cov*)

  Statistics of junction-scores defined in Section 2.3, which is the sum of scores of all transcript fragments that contain junction j and are compatible with p, i.e., $J(j, p) := \sum_{t:j\in t \text{ and } t\sim p} score(t)$.
- Junction coverage statistics:

  (*min_junc_cov*, *max_junc_cov*, *median_junc_cov*, *mean_junc_cov*, *std_junc_cov*)

  Statistics of junction-scores regardless of compatibility, i.e., $J'(j, p) := \sum_{t:j\in t} score(t)$.
- *extendable_score*: Cumulative junction scores for potential left/right path extensions.
- *cells_support*: Number of cells supporting path p.
- *ratio_cells_support*: Proportion of total cells supporting path p.
- *ratio_cells_fl_over_sample*: Proportion of cells supporting all junctions in p.
- *ratio_cells_fl_over_cells_support*: Proportion of full-length support among supporting cells.
- Cell support statistics:

  (*min_cell_junc*, *max_cell_junc*, *median_cell_junc*, *mean_cell_junc*, *std_cell_junc*)

  Statistics describing the number of cells supporting individual junctions $j \in p$.
- *num_fragments*: Total number of fragments compatible with p.
- Fragment Connecting:

  (*min_frag_cov*, *max_frag_cov*, *median_frag_cov*, *mean_frag_cov*, *std_frag_cov*)

  Statistics describing fragment coverage along path p.

Cell-Specific Features ($n = 21$)

- *input_genes*: Estimated number of input gene loci in cell c.
- *input_transcripts*: Number of input transcripts in cell c.
- *output_transcripts*: Number of output transcripts in cell c.
- *cell_supporting_junctions*: Number of junctions $j \in p$ supported by c.
- *cell_ratio_supporting_junctions*: Proportion of junctions $j \in p$ is supported by c.

- Cell-specific compatible junction coverage:

  (*cell_min_comp_cov*, *cell_max_comp_cov*, *cell_median_comp_cov*, *cell_mean_comp_cov*, *cell_std_comp_cov*)

  Statistics of cell-specific junction-scores, i.e., $J(c,p,j) := \sum_{t:j\in t \text{ and } t\sim p \text{ and } t\in c} score(t)$.
- *cell_nonzero_comp_cov*: Number of junction $j \in p$ such that $J(c,p,j) \neq 0$.
- *cell_max_streak_comp_cov*: Maximum number of continuous non-zero $J(c,p,j)$ for all junctions $j \in p$.
- *cell_min_streak_comp_cov*:Minimum number of continuous non-zero $J(c,p,j)$ for all junctions $j \in p$.
- Cell-specific Junction coverage:

  (*cell_min_junc_cov*, *cell_max_junc_cov*, *cell_median_junc_cov*, *cell_mean_junc_cov*, *cell_std_junc_cov*)

  Statistics of cell-specific junction-scores regardless of compatibility, i.e., $J'(c,p,j) := \sum_{t:j\in t \text{ and } t\in c} score(t)$.
- *cell_nonzero_junc_cov*: Number of junction $j \in p$ such that $J'(c,p,j) \neq 0$.
- *cell_max_streak_junc_cov*: Maximum number of continuous non-zero $J'(c,p,j)$ for all junctions $j \in p$.
- *cell_min_streak_junc_cov*: Minimum number of continuous non-zero $J'(c,p,j)$ for all junctions $j \in p$.

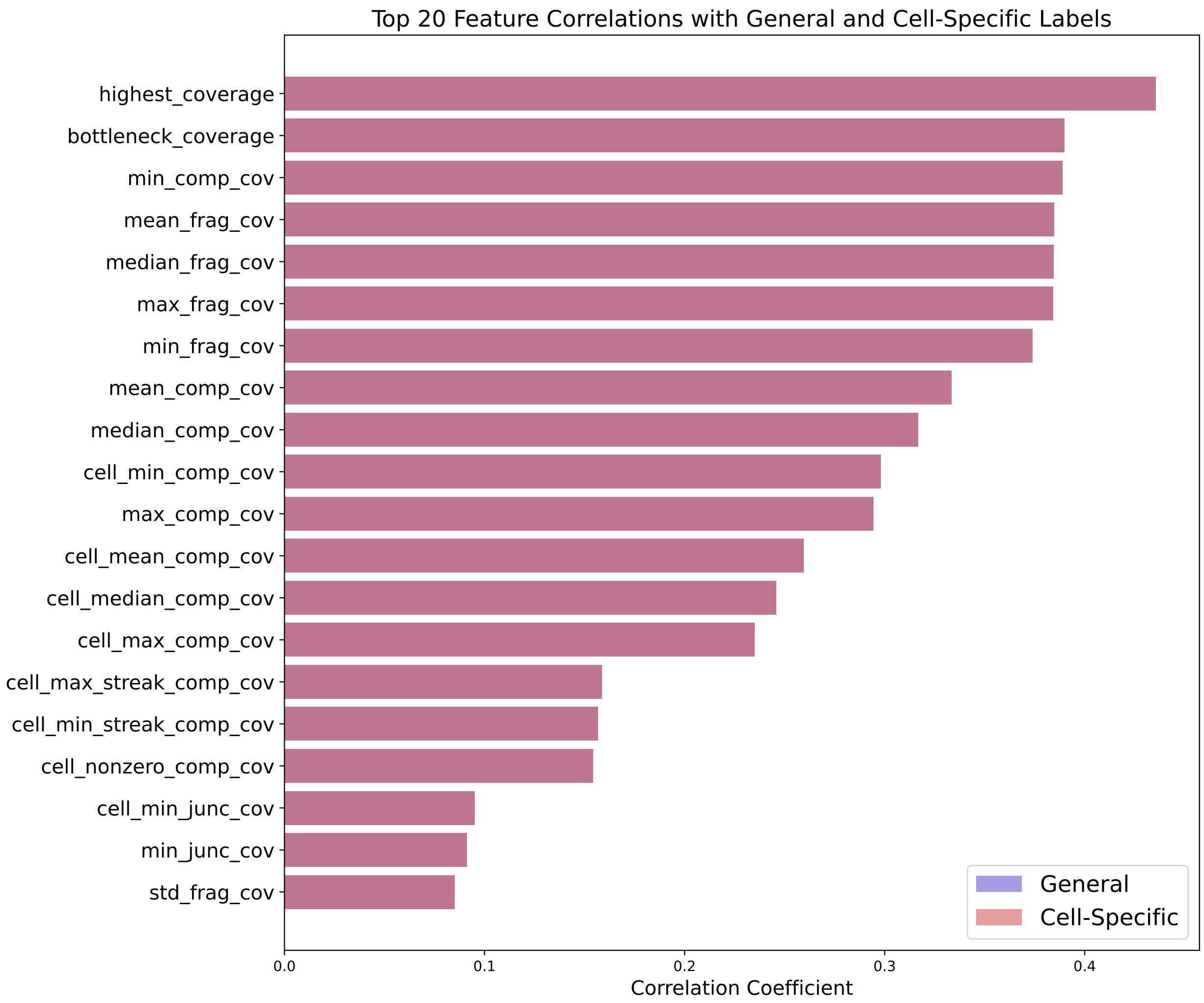

Supplementary Figure 1: Top 20 Feature Correlations with General and Cell-Specific Labels in the HEK293T Dataset. For the real datasets, the general and cell-specific labels fully coincide, resulting in identical correlations. (This is because, for real data, the ground-truth used for training both models are the same. Note that, for simulated data, they are different; see Supplementary Figure 3.) Among the three feature categories described in section "Scoring Assembled Full-length Transcripts (Feature Engineering)", the "Junction Coverage Features" exhibit the strongest positive correlation with transcript correctness. Notably, the feature *bottleneck_coverage*, which measures the minimum junction support along a transcript candidate, ranks among the highest correlated features. This indicates the intuition behind using the *bottleneck_coverage* score to select high-confidence candidate transcripts in the pre-sampling stage.

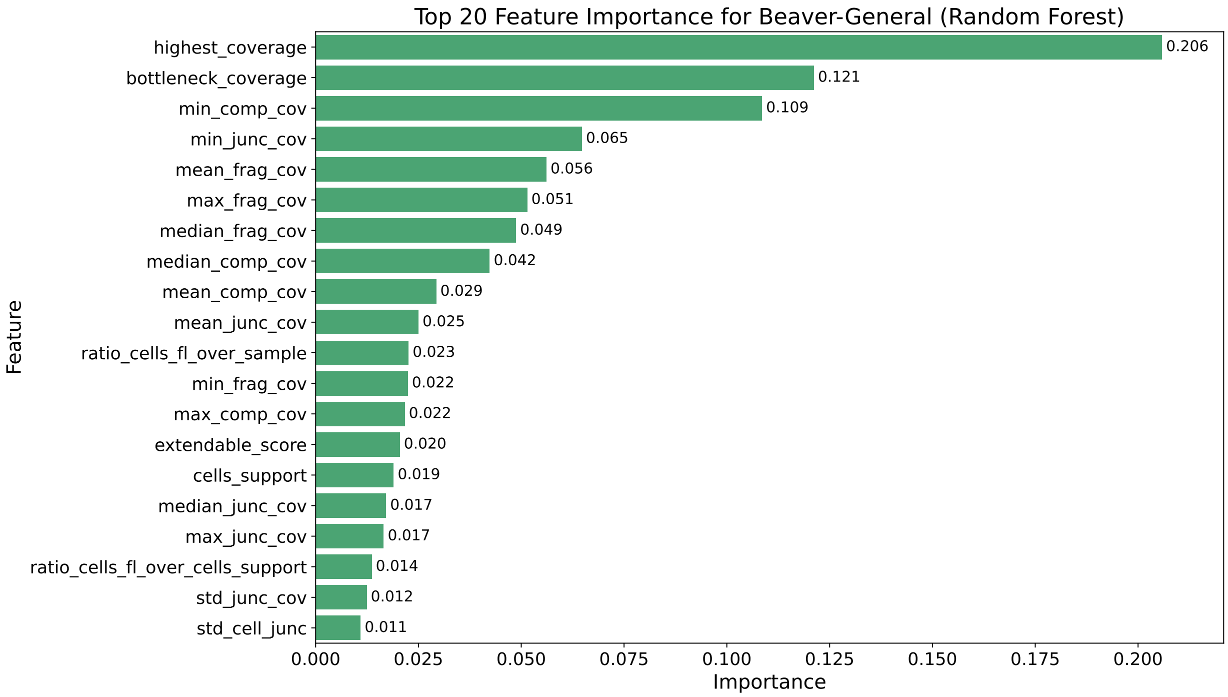

Supplementary Figure 2: Top 20 Feature Importance for Beaver-General in the HEK293T Dataset. The features identified as most important by the Beaver-General random forest model largely align with the features highly correlated with transcript correctness (see Supplementary Figure 1). An interesting example is *ratio_cells_fl_over_sample*, defined as the proportion of cells that support all junctions of a candidate transcript. Although this feature does not rank highly in correlation analysis, it emerges as important in the random forest model. A possible explanation is that this feature's significance primarily lies in distinguishing the extreme cases (value of 0 or not): a candidate transcript supported fully by no cell might indicate an artificial merging event, whereas full support from multiple cells strongly suggests transcript correctness.

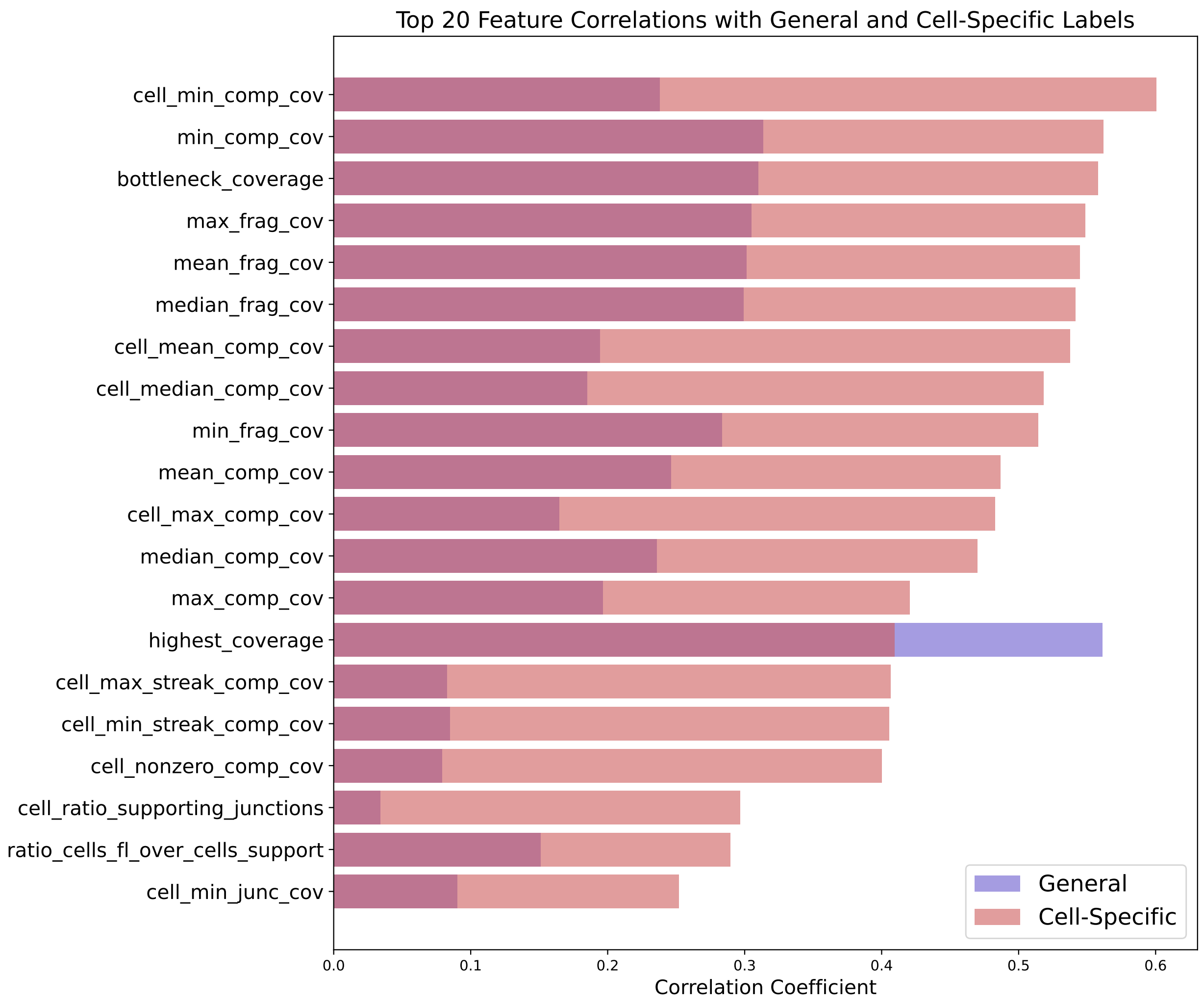

Supplementary Figure 3: Top 20 Feature Correlations with General and Cell-Specific Labels in the Simulated HEK293T-Sim Dataset. In simulated datasets, general and cell-specific labels differ due to the availability of exact cell-level ground truth. Consequently, features exhibit distinct correlations depending on the label type. Specifically, features prefixed with "cell_", which are designed as cell-specific features, show higher correlations with cell-specific labels but lower correlations with general labels, under expectations. Moreover, certain general features shared by both Beaver-General and Beaver-Specific, such as *highest_coverage*, display varying degrees of correlation. This variability highlights the rationale behind incorporating general features into both models, leveraging their predictive power differently in each context.

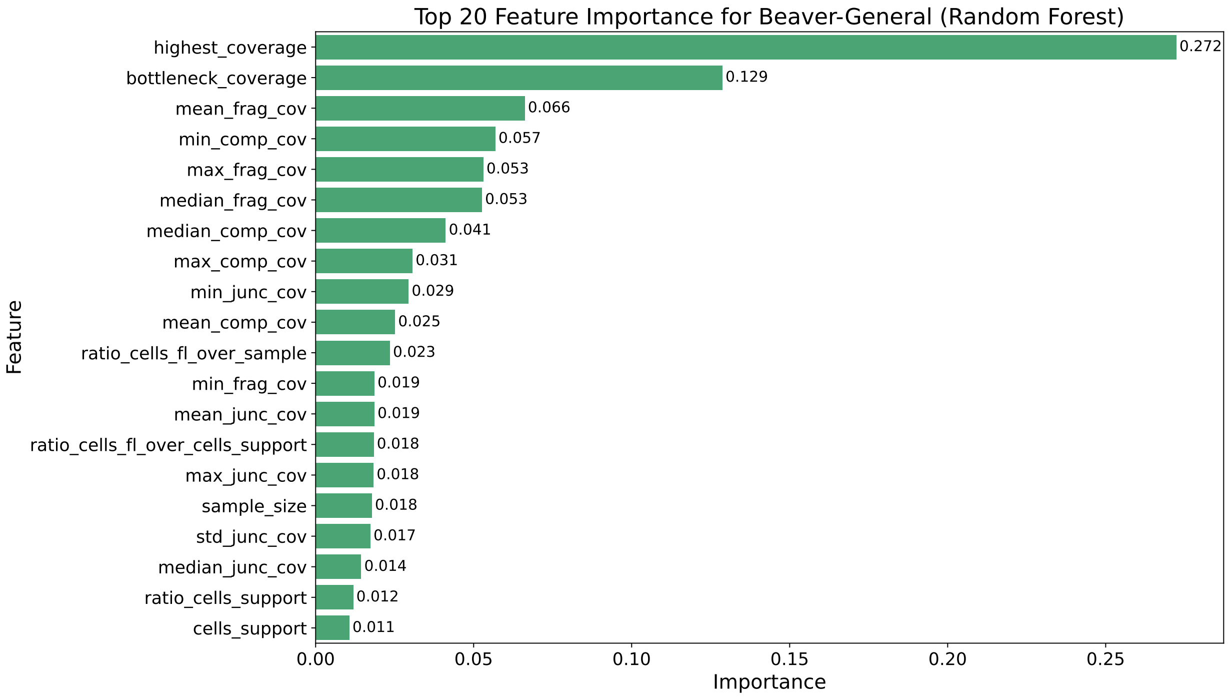

Supplementary Figure 4: Top 20 Feature Importance for Beaver-General in the HEK293T-Sim Dataset.

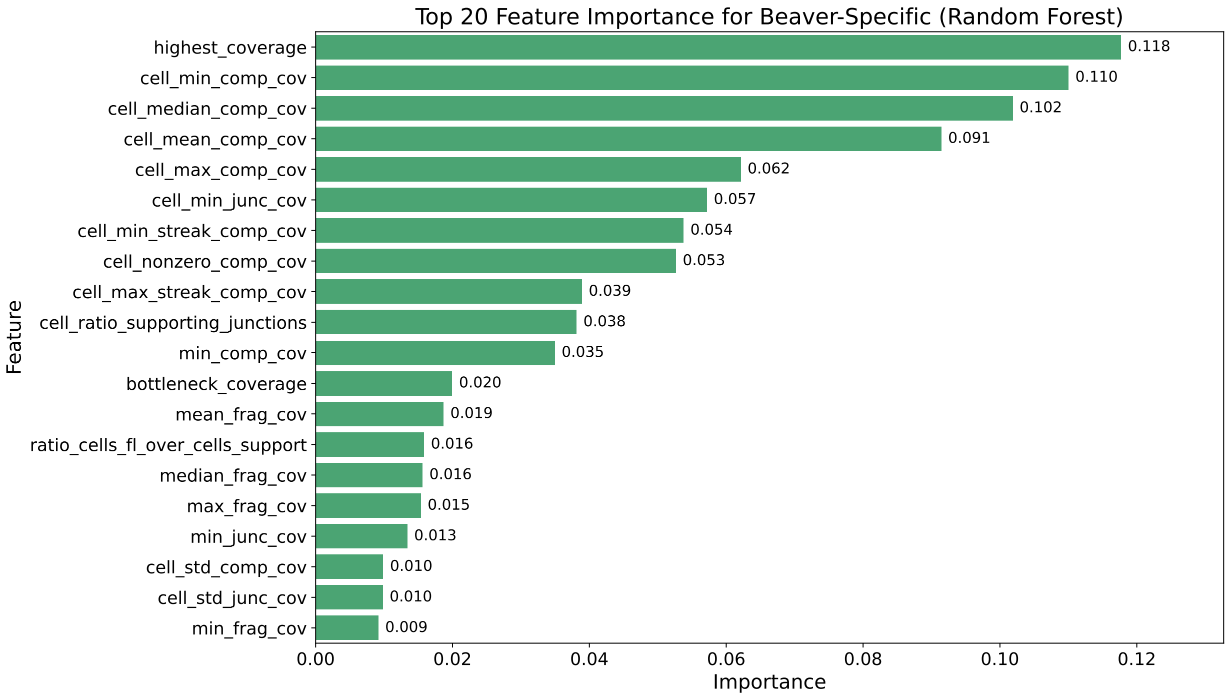

Supplementary Figure 5: Top 20 Feature Importance for Beaver-Specific in the HEK293T-Sim Dataset. In contrast to the Beaver-General, the Beaver-Specific model's feature importance rankings are dominated by cell-specific features. This clearly demonstrates that our carefully designed cell-specific feature engineering enhances the model's ability to accurately assign transcripts at the single-cell resolution.

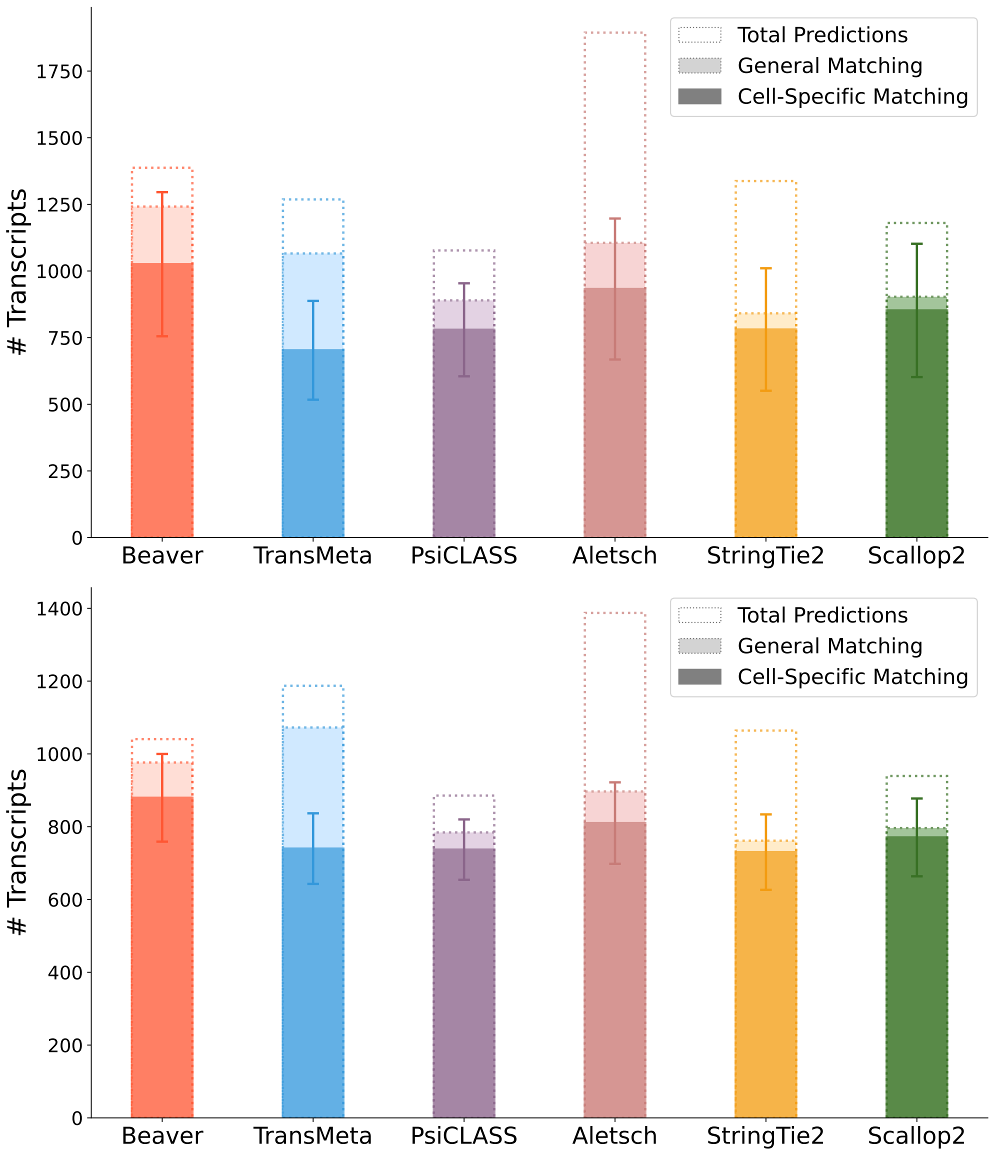

Supplementary Figure 6: Comparison of "General" versus "Cell-Specific" matching counts across assemblers in simulated datasets. Upper: HEK293T-Sim Dataset ($n = 192$); Lower: Fibroblast-Sim Dataset ($n = 369$).

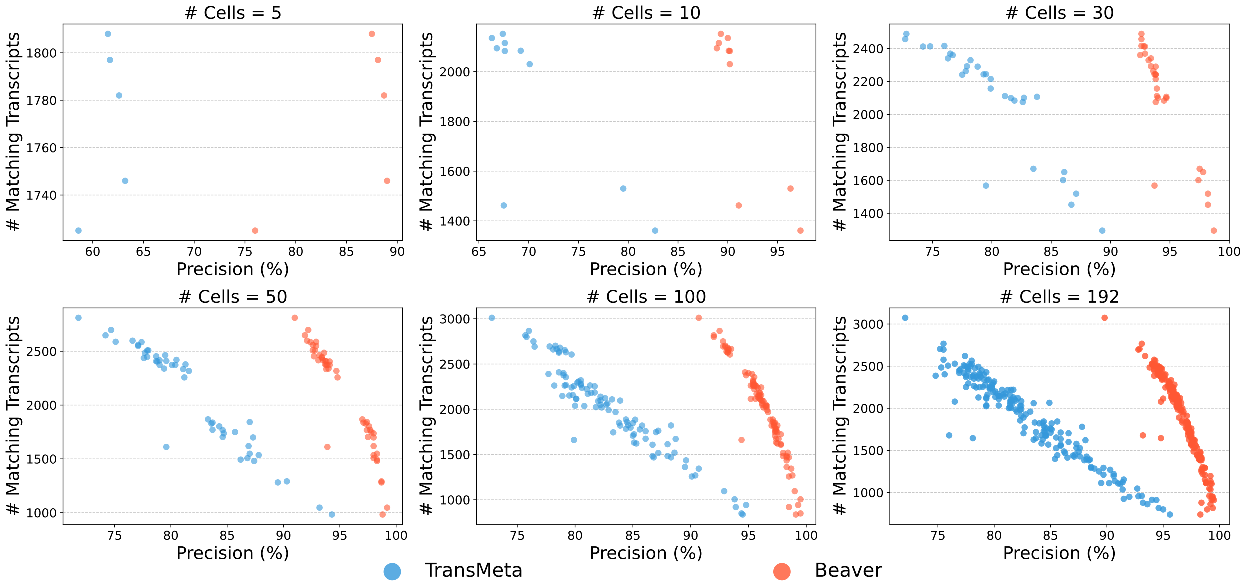

Supplementary Figure 7: Comparison of adjusted precision between Beaver and TransMeta across varying cell population sizes ($n = 5, 10, 30, 50, 100, 192$) in the HEK293T dataset. Each data point represents an individual cell.

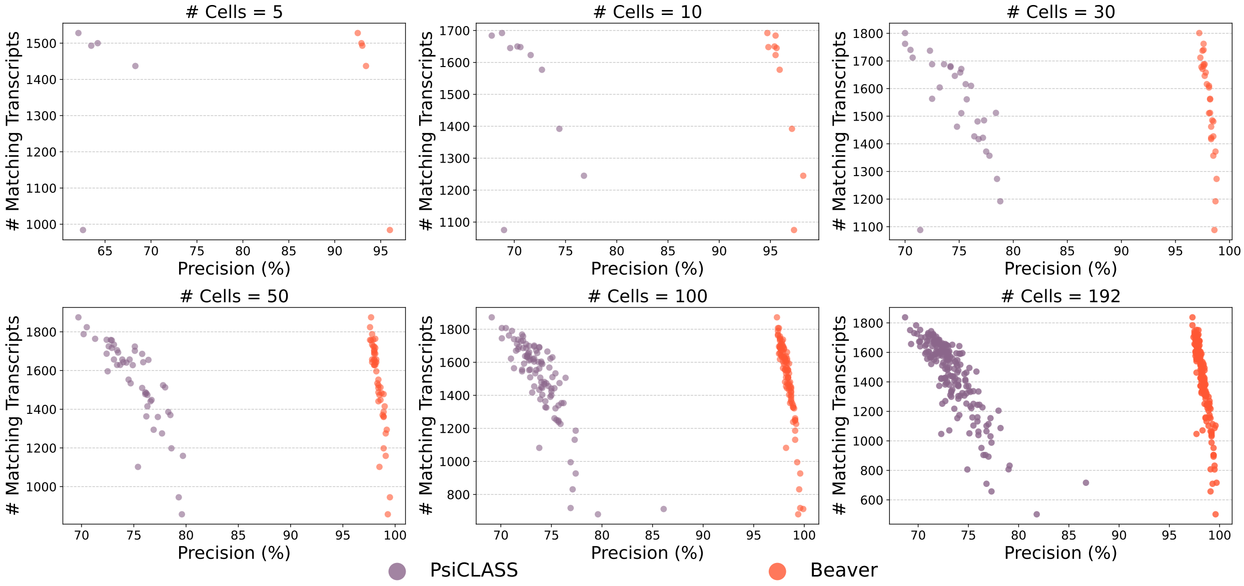

Supplementary Figure 8: Comparison of adjusted precision between Beaver and PsiCLASS across varying cell population sizes ($n = 5, 10, 30, 50, 100, 192$) in the HEK293T dataset. Each data point represents an individual cell.

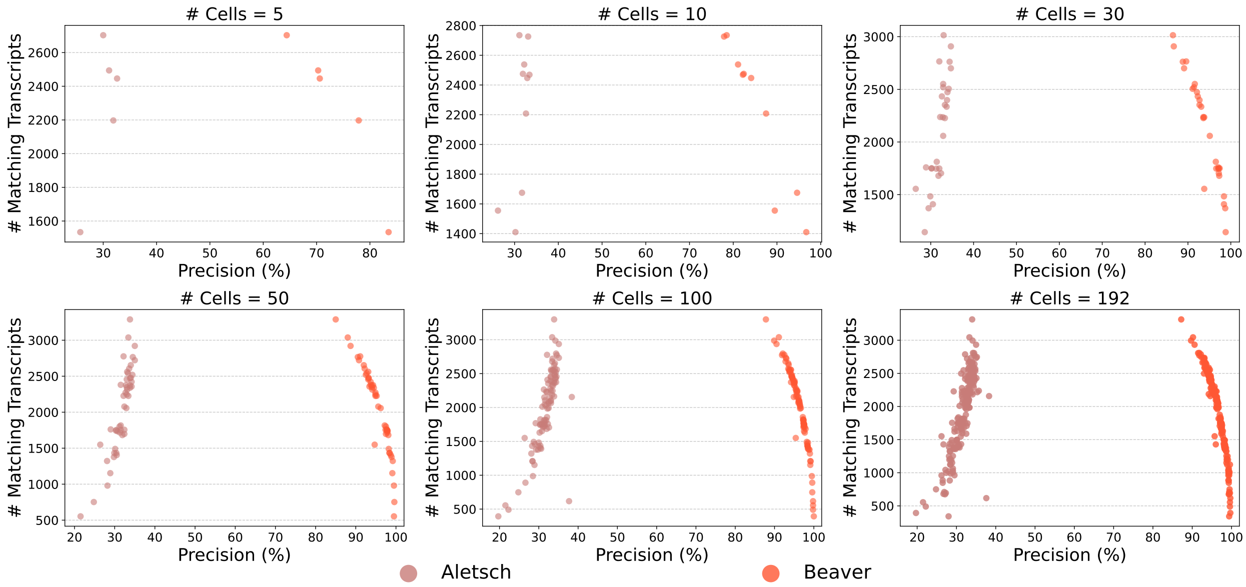

Supplementary Figure 9: Comparison of adjusted precision between Beaver and Aletsch across varying cell population sizes ($n = 5, 10, 30, 50, 100, 192$) in the HEK293T dataset. Each data point represents an individual cell.

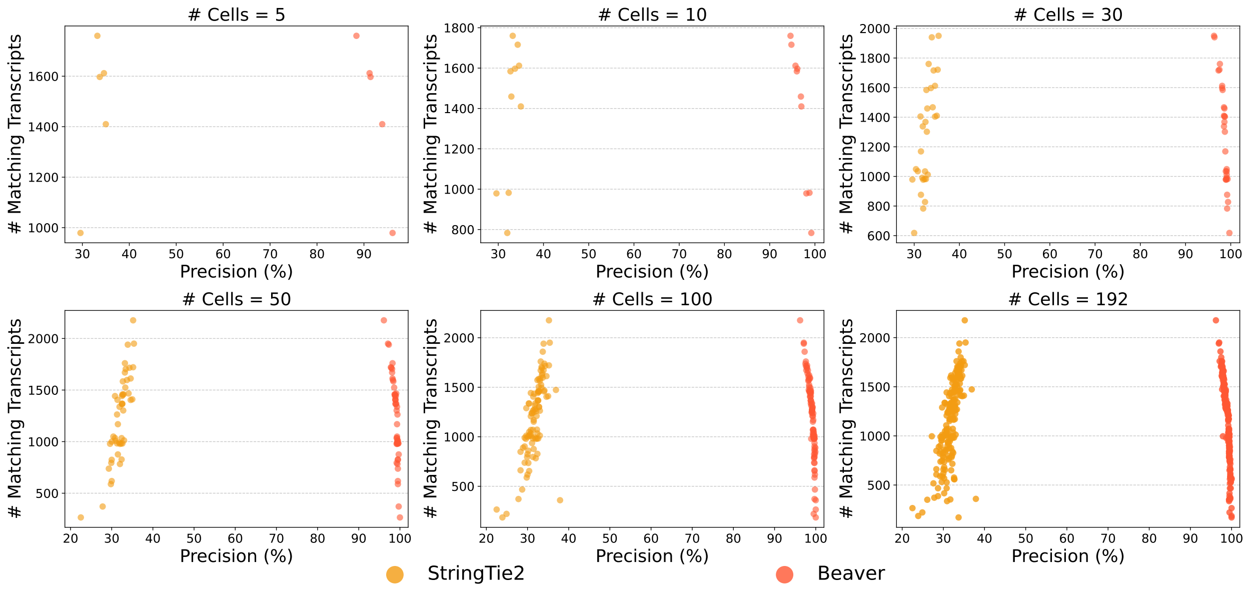

Supplementary Figure 10: Comparison of adjusted precision between Beaver and StringTie2 across varying cell population sizes ($n = 5, 10, 30, 50, 100, 192$) in the HEK293T dataset. Each data point represents an individual cell.

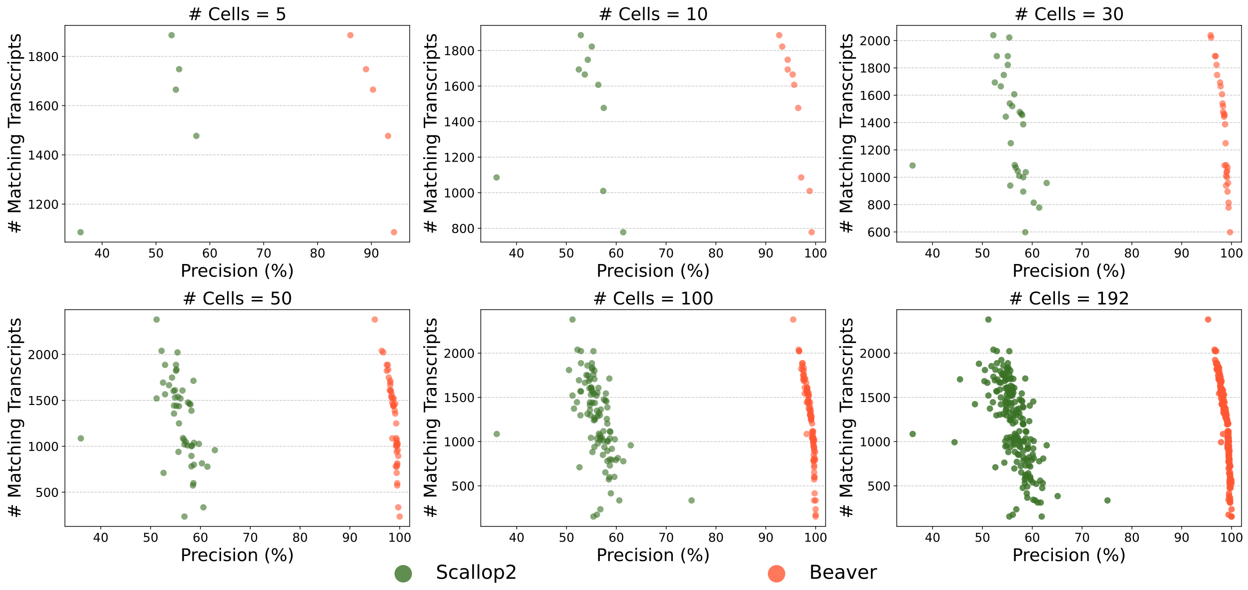

Supplementary Figure 11: Comparison of adjusted precision between Beaver and Scallop2 across varying cell population sizes ($n = 5, 10, 30, 50, 100, 192$) in the HEK293T dataset. Each data point represents an individual cell.

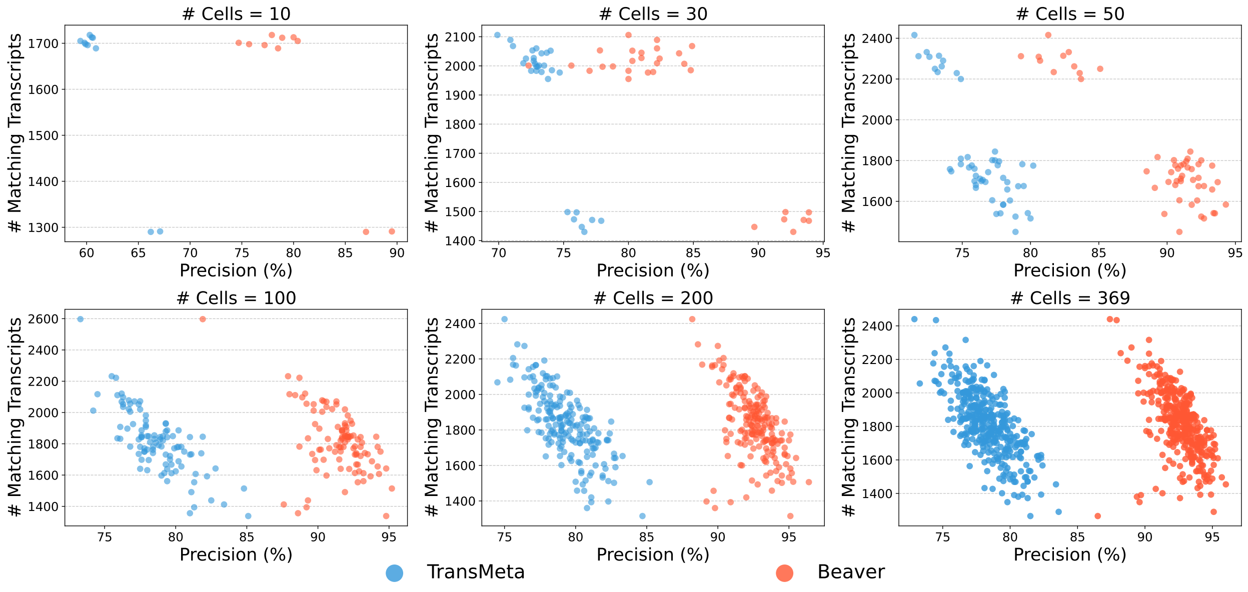

Supplementary Figure 12: Comparison of adjusted precision between Beaver and TransMeta across varying cell population sizes ($n = 10, 30, 50, 100, 200, 369$) in the Mouse-Fibroblast dataset. Each data point represents an individual cell.

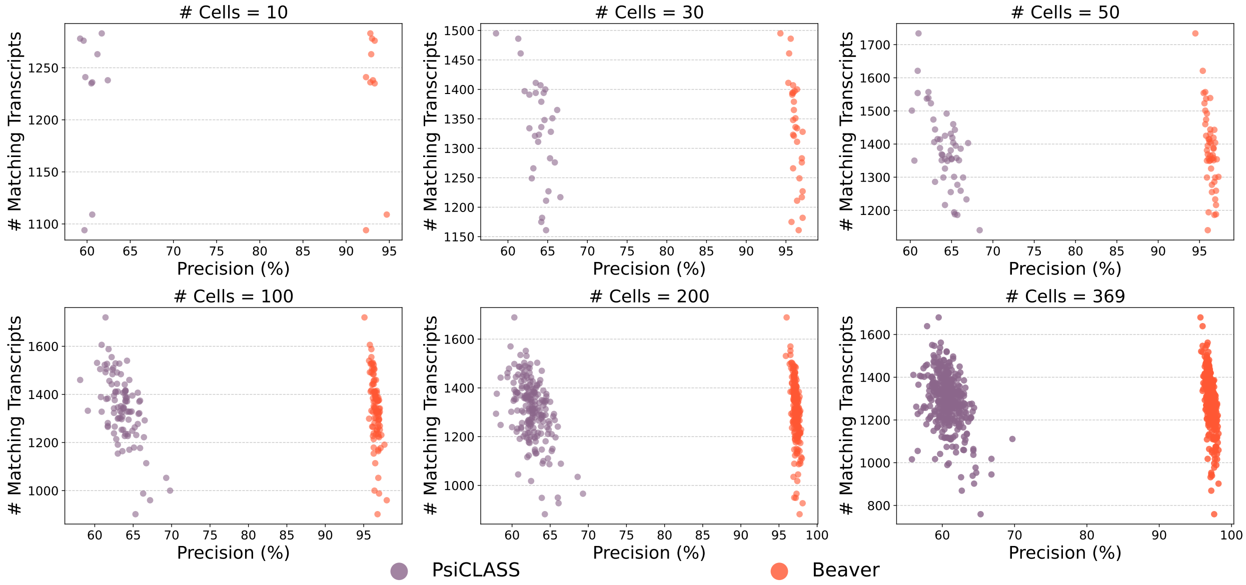

Supplementary Figure 13: Comparison of adjusted precision between Beaver and PsiCLASS across varying cell population sizes ($n = 10, 30, 50, 100, 200, 369$) in the Mouse-Fibroblast dataset. Each data point represents an individual cell.

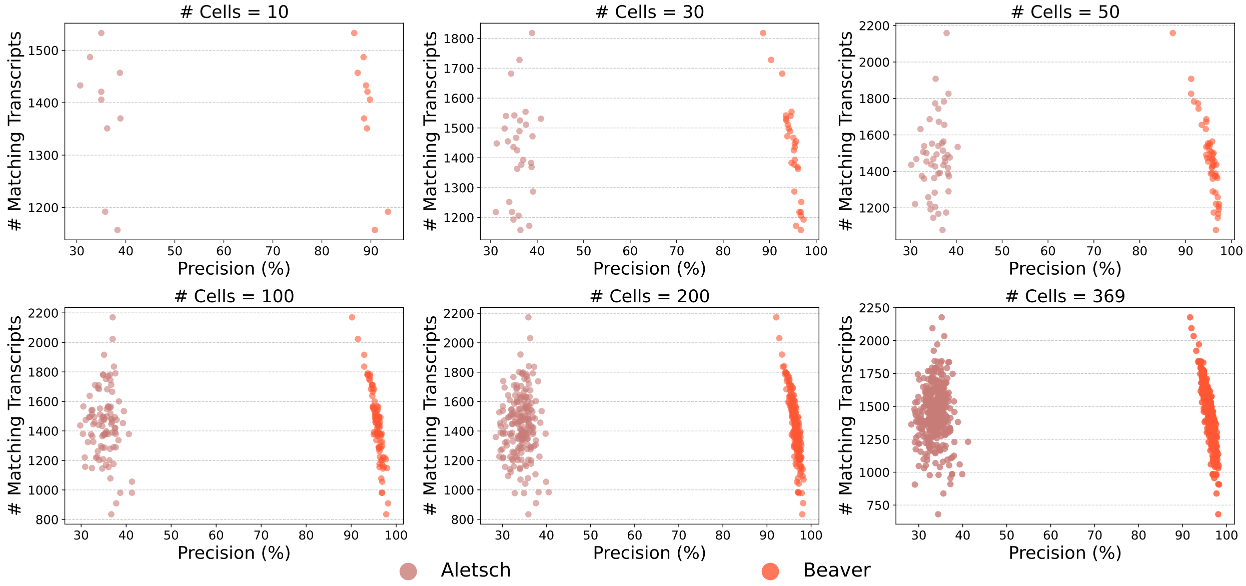

Supplementary Figure 14: Comparison of adjusted precision between Beaver and Aletsch across varying cell population sizes ($n = 10, 30, 50, 100, 200, 369$) in the Mouse-Fibroblast dataset. Each data point represents an individual cell.

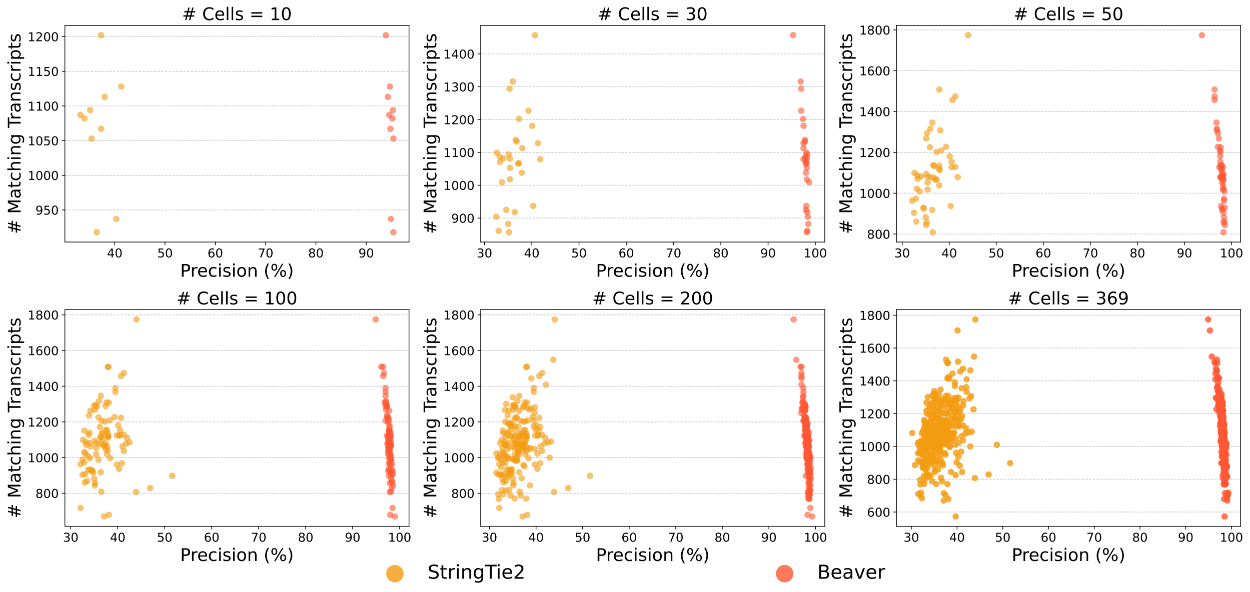

Supplementary Figure 15: Comparison of adjusted precision between Beaver and StringTie2 across varying cell population sizes ($n = 10, 30, 50, 100, 200, 369$) in the Mouse-Fibroblast dataset. Each data point represents an individual cell.

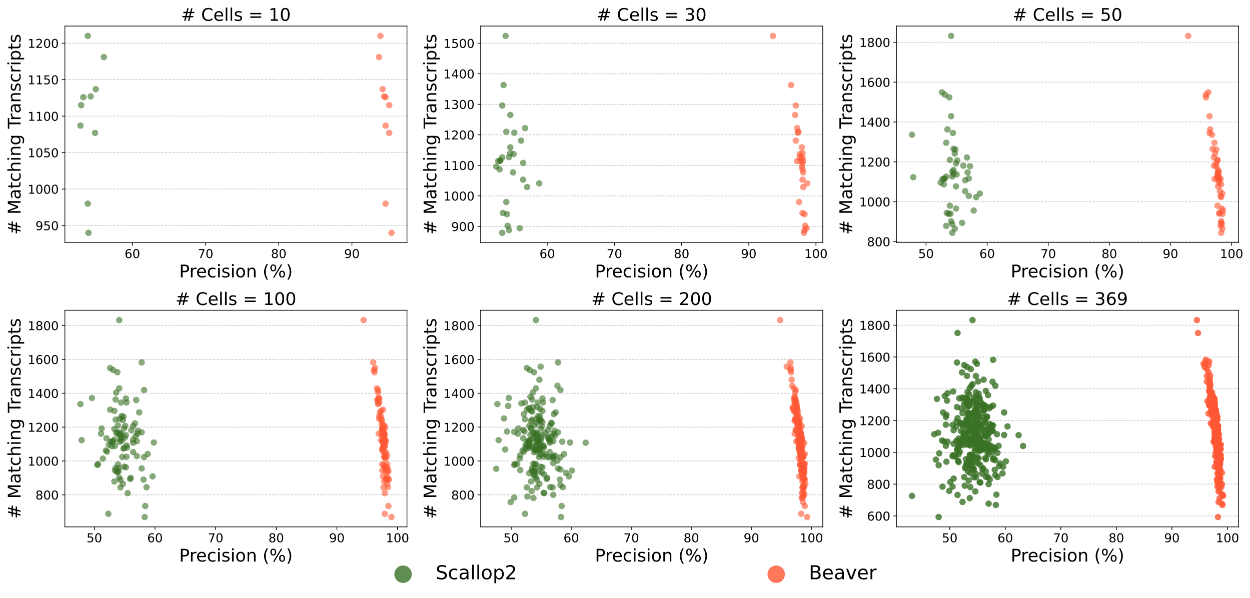

Supplementary Figure 16: Comparison of adjusted precision between Beaver and Scallop2 across varying cell population sizes ($n = 10, 30, 50, 100, 200, 369$) in the Mouse-Fibroblast dataset. Each data point represents an individual cell.

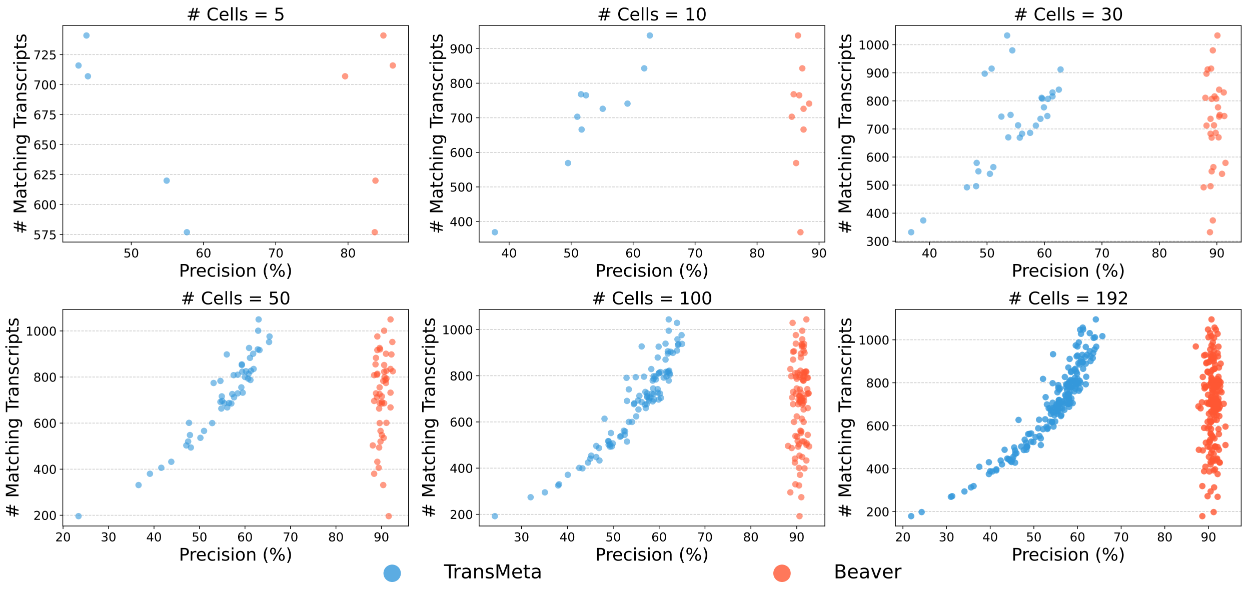

Supplementary Figure 17: Comparison of adjusted precision between Beaver and TransMeta across varying cell population sizes ($n = 5, 10, 30, 50, 100, 192$) in the simulated HEK293T-Sim dataset. Each data point represents an individual cell.

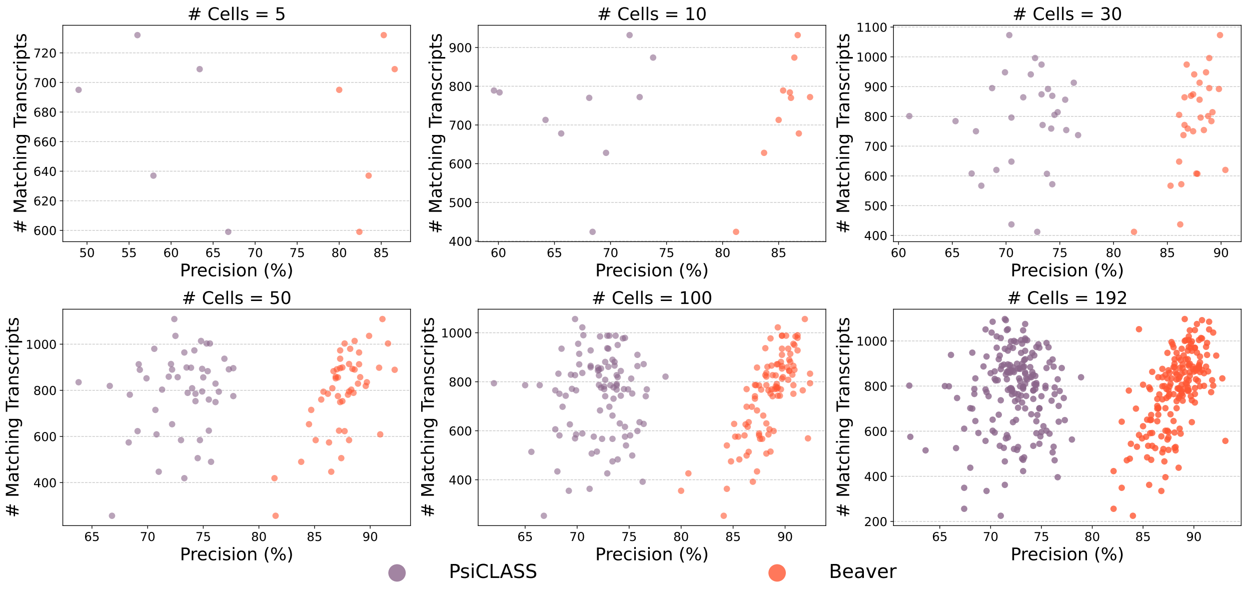

Supplementary Figure 18: Comparison of adjusted precision between Beaver and PsiCLASS across varying cell population sizes ($n = 5, 10, 30, 50, 100, 192$) in the simulated HEK293T-Sim dataset. Each data point represents an individual cell.

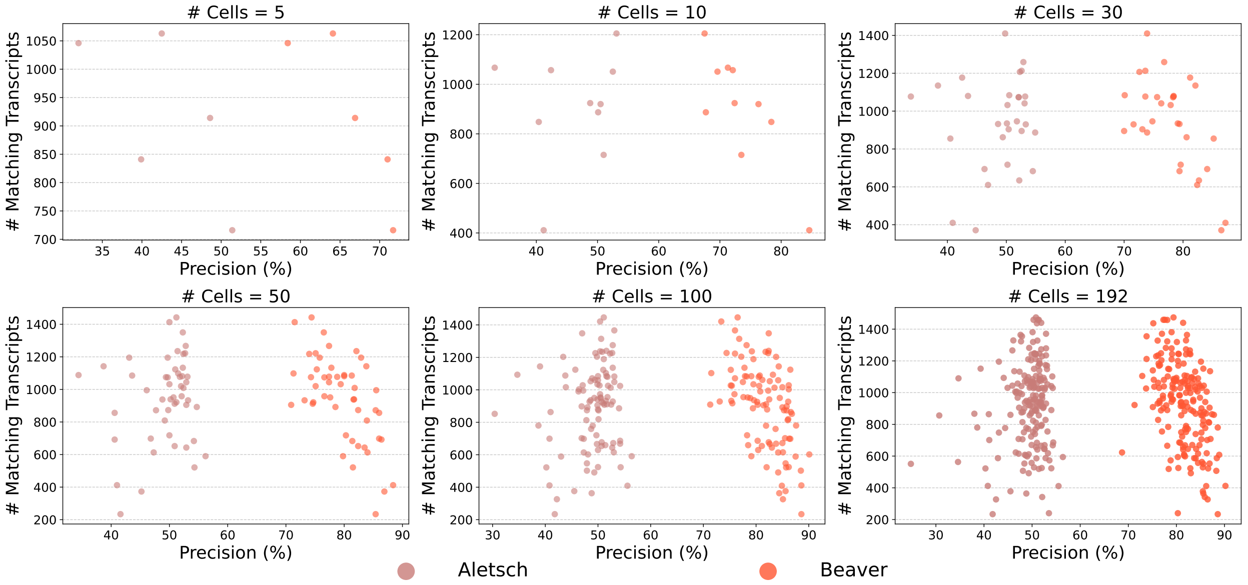

Supplementary Figure 19: Comparison of adjusted precision between Beaver and Aletsch across varying cell population sizes ($n = 5, 10, 30, 50, 100, 192$) in the simulated HEK293T-Sim dataset. Each data point represents an individual cell.

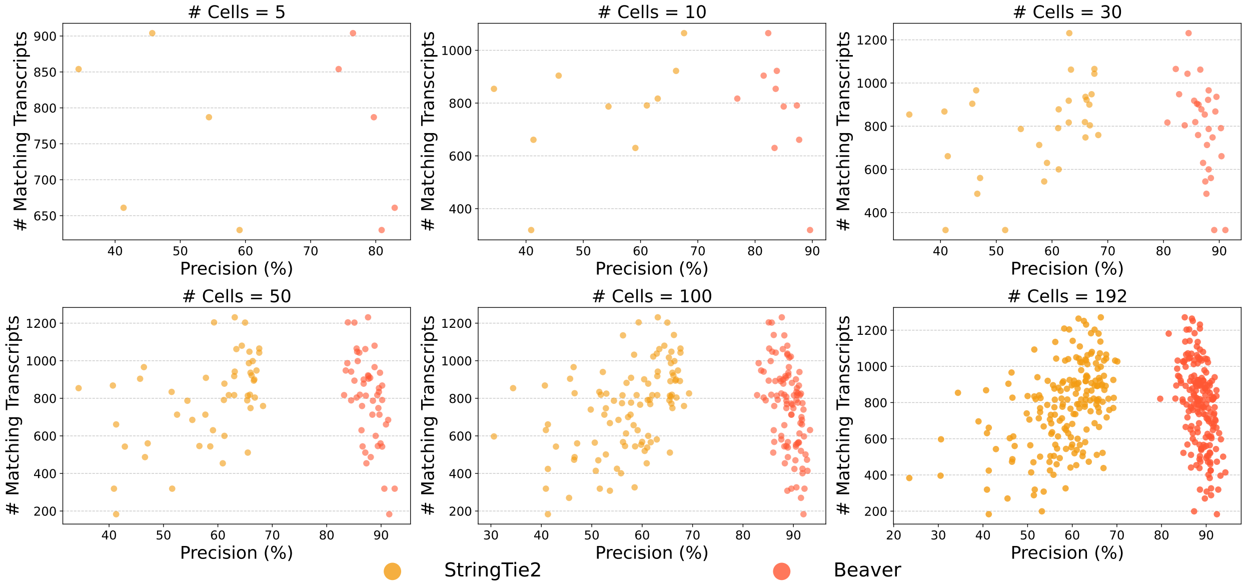

Supplementary Figure 20: Comparison of adjusted precision between Beaver and StringTie2 across varying cell population sizes ($n = 5, 10, 30, 50, 100, 192$) in the simulated HEK293T-Sim dataset. Each data point represents an individual cell.

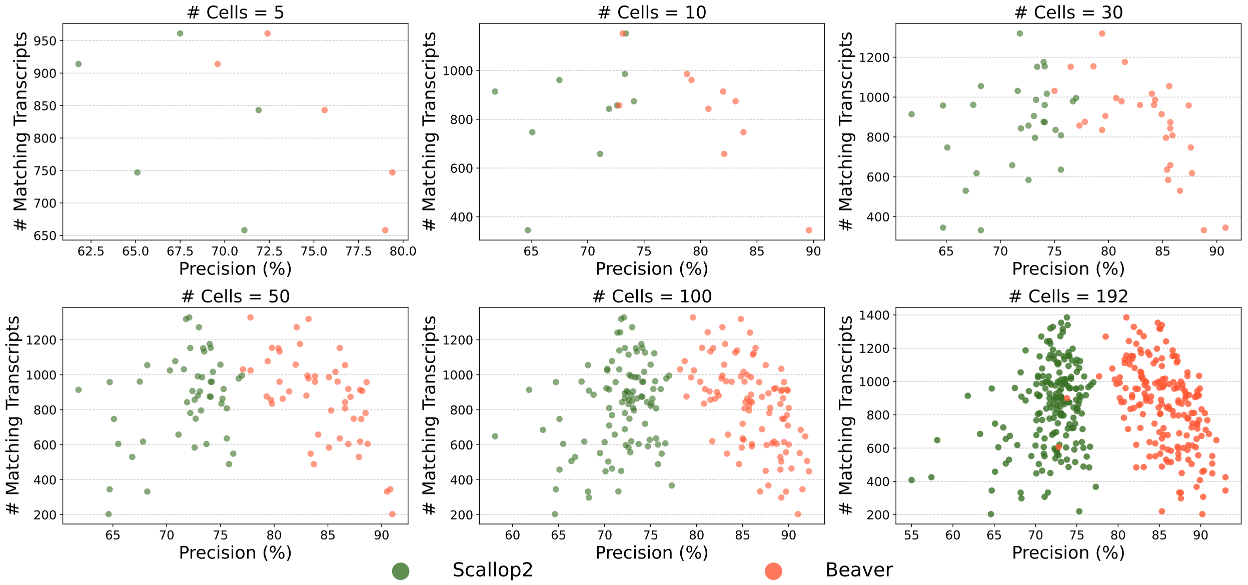

Supplementary Figure 21: Comparison of adjusted precision between Beaver and Scallop2 across varying cell population sizes ($n = 5, 10, 30, 50, 100, 192$) in the simulated HEK293T-Sim dataset. Each data point represents an individual cell.

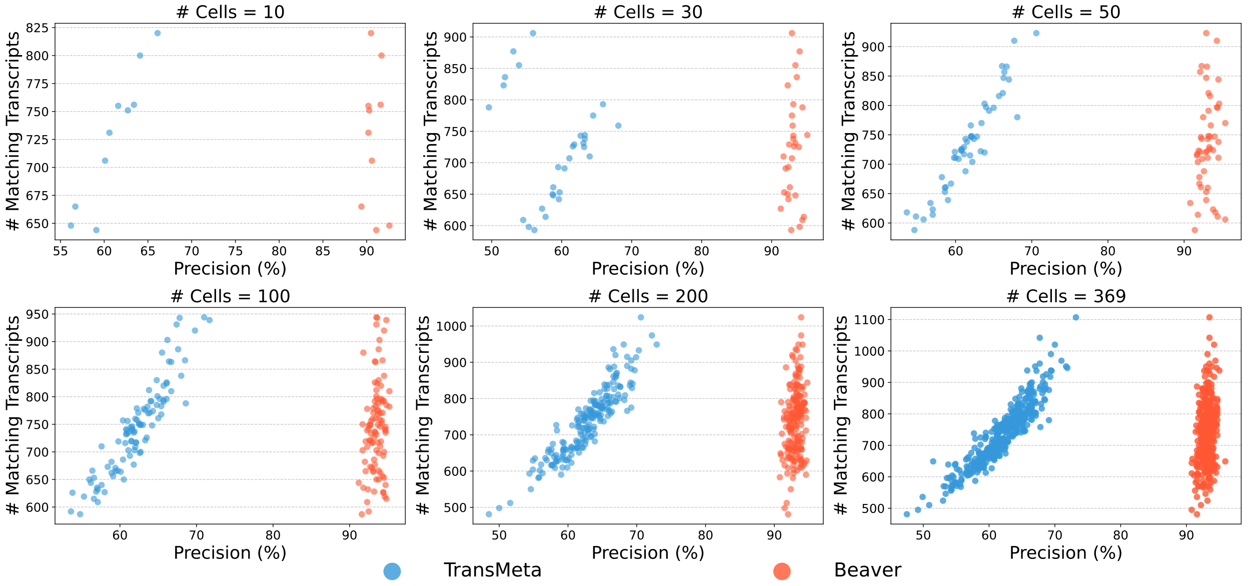

Supplementary Figure 22: Comparison of adjusted precision between Beaver and TransMeta across varying cell population sizes ($n = 10, 30, 50, 100, 200, 369$) in the simulated Mouse-Fibroblast-Sim dataset. Each data point represents an individual cell.

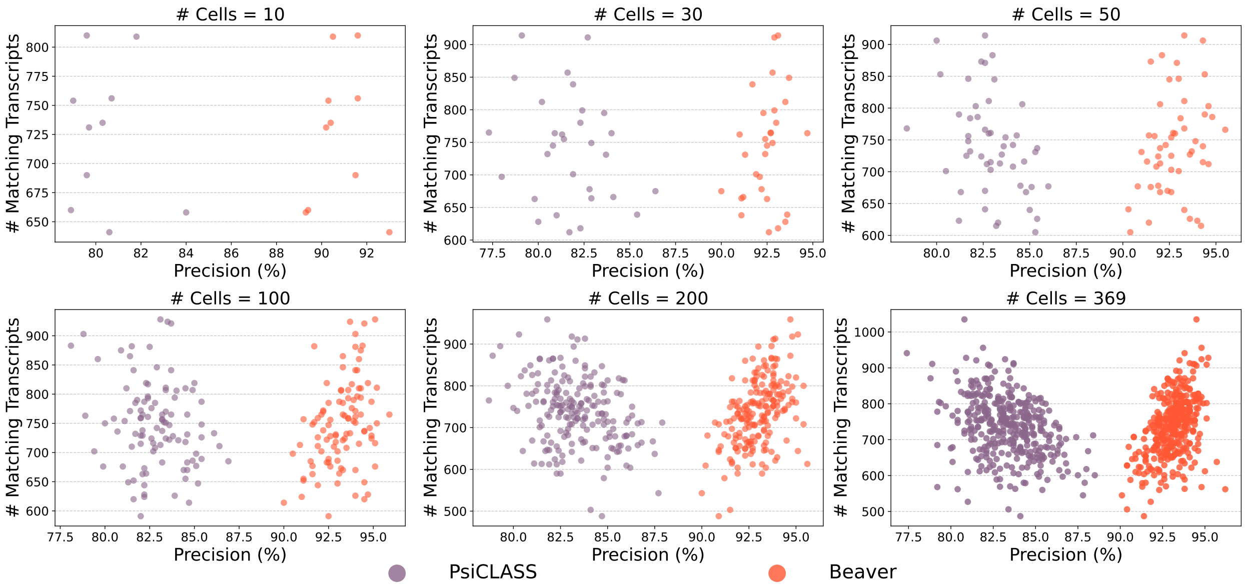

Supplementary Figure 23: Comparison of adjusted precision between Beaver and PsiCLASS across varying cell population sizes ($n = 10, 30, 50, 100, 200, 369$) in the simulated Mouse-Fibroblast-Sim dataset. Each data point represents an individual cell.

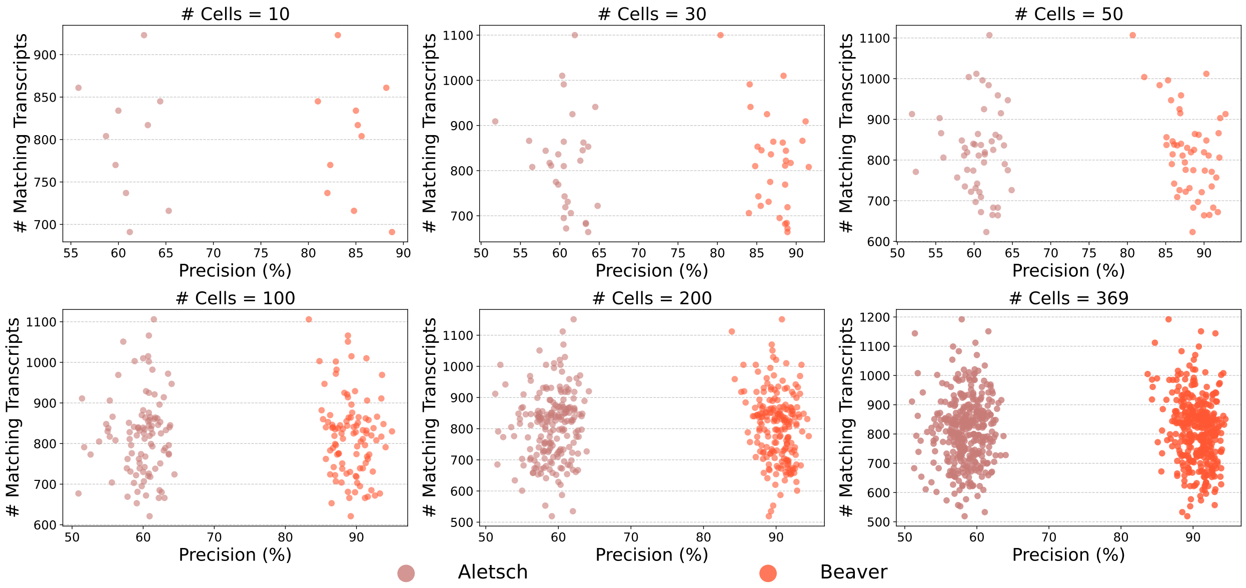

Supplementary Figure 24: Comparison of adjusted precision between Beaver and Aletsch across varying cell population sizes ($n = 10, 30, 50, 100, 200, 369$) in the simulated Mouse-Fibroblast-Sim dataset. Each data point represents an individual cell.

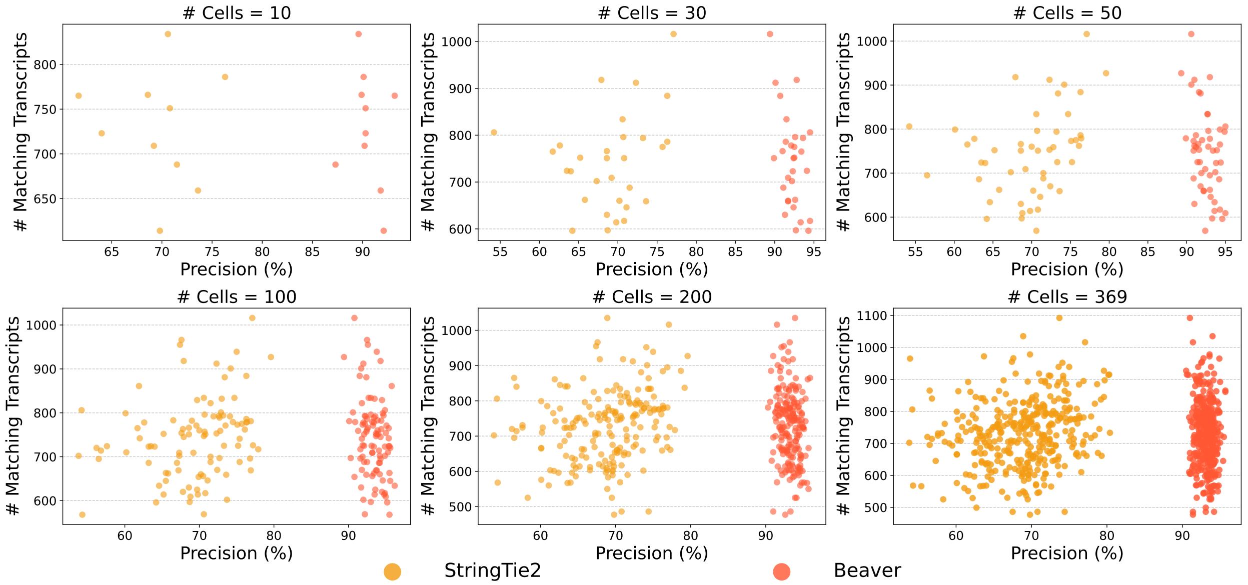

Supplementary Figure 25: Comparison of adjusted precision between Beaver and StringTie2 across varying cell population sizes ($n = 10, 30, 50, 100, 200, 369$) in the simulated Mouse-Fibroblast-Sim dataset. Each data point represents an individual cell.

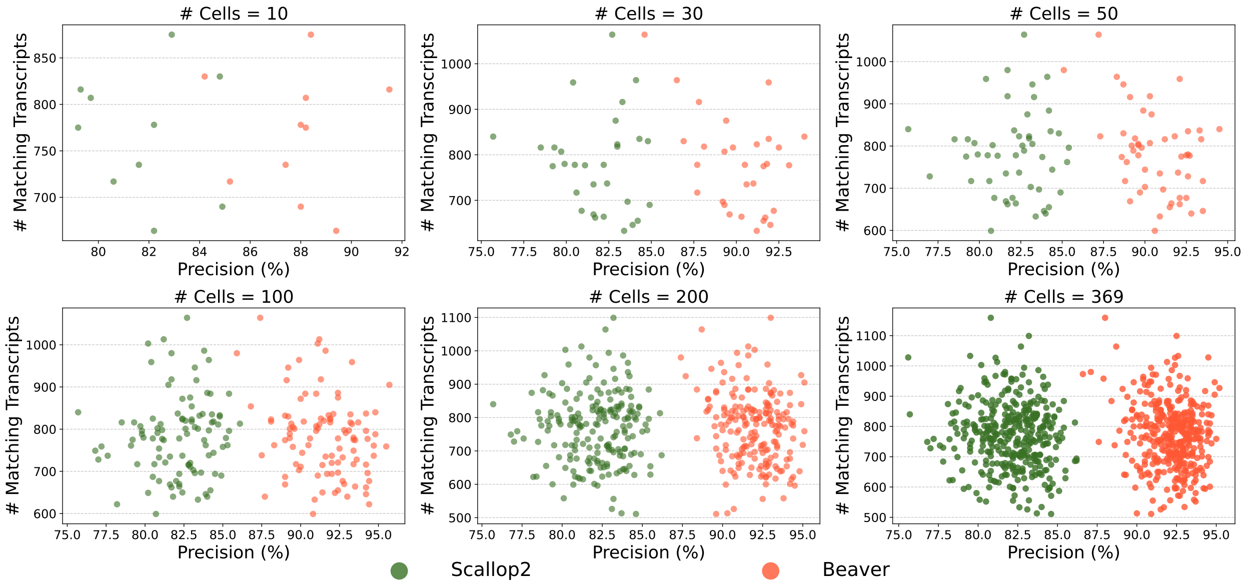

Supplementary Figure 26: Comparison of adjusted precision between Beaver and Scallop2 across varying cell population sizes ($n = 10, 30, 50, 100, 200, 369$) in the simulated Mouse-Fibroblast-Sim dataset. Each data point represents an individual cell.

Supplementary Table 1: Description of HEK293T and Mouse-Fibroblast datasets. Both datasets were generated with the Smart-seq3 protocol.

Dataset Name	Species	Cell Type	#Cells	Read Count	Base Count	Accession ID
HEK293T	*Homo sapiens*	Kidney epithelial	192	235,242,464	15,646,451,072	ERR4018555
Mouse-Fibroblast	*Mus musculus*	Tail fibroblast	369	1,138,473,032	164,508,417,618	ERR3835351
